# Study of the Grape Cryo-Maceration Process at Different Temperatures

**DOI:** 10.3390/foods7070107

**Published:** 2018-07-06

**Authors:** Daniele Naviglio, Andrea Formato, Giampiero Scaglione, Domenico Montesano, Arcangelo Pellegrino, Francesco Villecco, Monica Gallo

**Affiliations:** 1 Department of Chemical Sciences, University of Naples Federico II, via Cinthia; Monte S. Angelo Complex, 80126 Naples, Italy; naviglio@unina.it; 2 Department of Agricultural Science, University of Naples Federico II, Via Università 100, Portici, 80045 Naples, Italy; andrea.formato@unina.it (A.F.); giampiero.scaglione@unina.it (G.S.); 3 Department of Pharmaceutical Sciences, Food Science and Nutrition Section, University of Perugia, via S. Costanzo 1, 06126 Perugia, Italy; domenico.montesano@unipg.it; 4 Department of Industrial Engineering, University of Salerno, Via Giovanni Paolo II 132, 84084 Fisciano, Italy; apellegrino@unisa.it (A.P.); fvillecco@unisa.it (F.V.); 5 Department of Molecular Medicine and Medical Biotechnology, University of Naples Federico II, via Pansini 5, 80131 Naples, Italy

**Keywords:** Chardonnay grapes, cryo-maceration, liquid carbon dioxide, polyphenols, numerical simulation

## Abstract

This research aimed to determine the effects of cryo-maceration at different temperatures on polyphenol content during the winemaking process of Chardonnay wine. Samples of Chardonnay grapes were subjected to rapid cooling processes by direct injection of liquid CO_2_ to obtain final temperatures of 10.0, 8.0, 6.0 and 4.0 °C and yield different batches of grape mash. Subsequently, each batch underwent the winemaking process to produce four different wines. The wines obtained were characterized by chemical analyses. We observed higher extraction of polyphenolic compounds with low-temperature cold maceration, particularly when the temperature was reduced from 10.0 to 6.0 °C. Conversely, when the temperature was reduced below 6.0 °C, the increase in polyphenol content in wine was negligible, whereas CO_2_ consumption increased. Furthermore, a numerical simulation was performed to determine the pipe length, *L*_0_, after which the temperature was constant. This condition is very important because it guarantees that after the length *L*_0_, the thermodynamic exchange between liquid CO_2_ and is complete, eliminating the possibility of liquid CO_2_ pockets in the cyclone.

## 1. Introduction

Studies of extraction methods and their conditions play a central role not only in the exhaustive recovery of bioactive compounds from natural matrices, but also in the preservation of these compounds in their native form by avoiding alterations [1,2]. In the last few years, inert gas has been widely used in winemaking processes to cool and protect musts from oxidation, with satisfactory results [3,4,5]. The cryo-maceration of grapes by the direct contact of mashed grapes with a cryogenic gas is a promising technical approach to winemaking [6,7]. This process allows the must to rapidly reach temperatures below 10.0 °C, thereby preventing an increase in the yeast population, alcoholic fermentation, and the loss of volatile aromatic compounds [8]. Pre-fermentative white grape cryo-maceration is normally used to enhance the varietal character of white wine by extraction of the skin flavour, and the grape skin contact is conducted in controlled conditions (time and temperature). Different tests reported in the literature have aimed at maintaining the pre-fermentation phase to prevent the initiation of undesired spontaneous fermentation rather than understanding the effects of cooling [9,10]. Cold pre-fermentation maceration is realized by submitting the mashed grapes to temperatures below 18.0–20.0 °C [11,12]. This process can be performed by controlling the temperatures of refrigerated containers, by flowing mashed grapes through tubular heat exchangers, or by direct contact of cryogenic gas with the mashed grapes. Previous experimental data have indicated that cold pre-fermentation maceration between 10 and 20 h and control of the temperature in an oxygen-poor atmosphere positively affects the extraction of aromatic and phenolic substances to increase the varietal aroma [13]. A considerable amount of gas evolves during the direct contact between the must and cryogen, which reduces the diffusion of atmospheric oxygen into the liquid phase, and thus decreases the oxidation of phenolic substances, aromatic compounds and particularly anthocyanins, which are responsible for the colour of red wines [14,15]. Grape skins, pulp and seeds contain a large amount of different phenolic compounds that are partially extracted during winemaking [16,17]. These phenolic compounds, together with those arising from chemical reactions occurring during the winemaking and/or ageing processes, affect the colour, taste and structure of wines [18,19]. Wine polyphenols have been studied extensively in relation to their protective effects against cardiovascular and degenerative diseases [20], certain types of cancers [21] and chronic inflammation and thrombosis [22]. Their chemical structures enable them to act as antioxidants to scavenge and neutralize free radicals [23]. Cryo-maceration equipment for fast cooling of mashed grapes by means of a cryogenic gas (liquid carbon dioxide, CO_2_) is already available on the market. A dosed quantity of liquid CO_2_ is directly injected in-line from a tank into the mashed grape flow, which is cooled to the pre-set desired temperature. The CO_2_ consumption depends on both the mass quantity to be cooled and the final temperature. Therefore, liquid CO_2_ consumption increases with increasing temperature difference (DT) in the process. Parenti et al. (2004) [24] reported that cold maceration with cryogenic gas increased the total polyphenol content of typical Tuscan red grapes of Chianti (Sangiovese), with cryo-maceration temperatures ranging from +5.0 to −5.0 °C. However, this process causes phase changes; in fact, at 0 °C and –5.0 °C, the aqueous substance of the mashed grape turned to ice, with consequent alterations in its physical-chemical properties. However, many producers use cryo-maceration processes with cryo-maceration temperatures as low as possible to avoid ice formation. For these types of plants, however, during the thermodynamic exchange between liquid CO_2_ and must, liquid CO_2_ pockets may form, which will cause ice formation in the cyclone (a condition to avoid). Therefore, this work evaluate cryo-maceration temperatures that inhibit (or almost inhibit) the fermentation process in an attempt to avoid phase variations that could affect the physicochemical characteristics of the mashed grape. Samples of Chardonnay grapes were analysed at different cryo-maceration temperatures (10.0, 8.0, 6.0, 4.0 °C) to evaluate the polyphenol content extracted without phase changes (ice formation) under the same operating conditions. Finally, for the considered process, a numerical simulation was performed to evaluate the pipe length *L*_0_ after which the temperature distribution is uniform and the thermodynamic exchange between liquid CO_2_ and must is complete, thereby avoiding liquid CO_2_ pockets.

## 2. Materials and Methods

### 2.1. Instrumentation and Reagents

Polyphenols were analysed with an HP 8452 Agilent UV-Vis spectrophotometer (Agilent Technologies, Santa Clara, CA, USA). The reagents *p*-(dimethylamino)cinnamaldehyde (*p*-DMACA), gallic acid and (+)-catechin were purchased from Fluka (Buchs, Switzerland), and the Folin-Ciocalteu reagent was from Merck (Darmstadt, Germany). All other employed reagents were of analytical grade.

Standards (−)-Epicatechin, (+)-catechin, (−)-epigallocatechin, and (−)-epigallocatechin gallate were purchased from Sigma Chemical (St. Louis, MO, USA).

**Figure 1:**
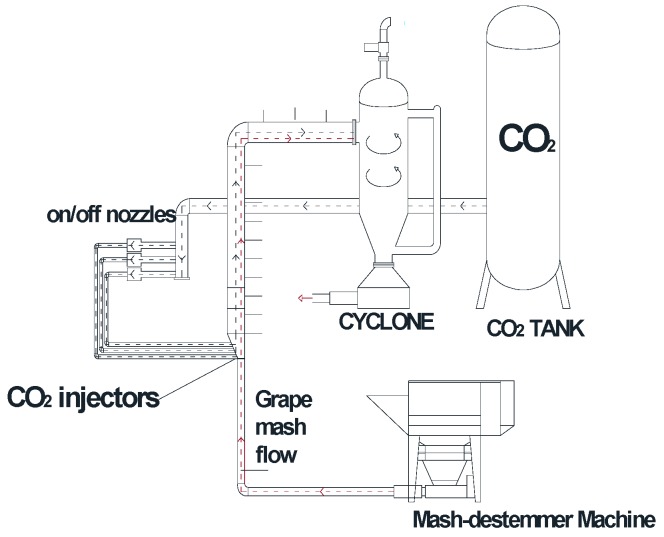
Grape cryomaceration equipment with liquid CO_2_.

The Chardonnay grapes used in this study were from Campania (Benevento, Italy). The grapes had the following characteristics: 10.5 ± 0.5 (*v*/*v*) sugar; 20.5 ± 0.5 °Brix; pH = 3.45 ± 0.05; total acidity 6.5 ± 0.5 (g/L). These values were considered optimal for the production of a good tasting Chardonnay wine. The grapes were harvested manually. After destalking and mashing, the mashed grapes were cooled rapidly, in only a few seconds, to the pre-set cryo-maceration temperature in specialized equipment using direct in-line liquid CO_2_ injection. The equipment consisted of a mash-destemmer machine whose final stage was connected to a thermally insulated AISI 304 steel principal inlet pipe with an internal diameter of 30 cm and a thickness of 3 mm; the mashed grapes flowed through this pipe. The liquid CO_2_ system consisted of a liquid CO_2_ tank (20 × 10^5^ Pa), liquid CO_2_ injectors, pipelines and nozzles (thermally insulated) for the direct injection of liquid CO_2_ into the mashed grapes. A cyclone was used for solid-gas separation. A control panel allowed the regulation of process data (i.e., levels, pressure and temperature) via a mono-type volumetric pump. A schematic representation of the equipment is shown in Figure 1. The equipment is generally used for cooling grapes at a rate of 20–25 ton/h, with an adjustable temperature range from 10.0 to 25.0 °C. Using the equipment shown in Figure 1, the mashed grape batches underwent the cryo-maceration process at temperatures of 10.0, 8.0, 6.0 and 4.0 °C, with a standard deviation of ± 0.5 °C.

### 2.2. HPLC Analysis of Polyphenols 

Chromatographic analyses were performed on an Hewlett-Packard (HP) 1100 Series HPLC (High Performance Liquid Chromatography) (Agilent Technologies, Santa Clara, CA, USA) equipped with an autosampler, quaternary pump, column heater, diode array detector, HP1046A fluorescence detector (Agilent Technologies, Santa Clara, CA, USA), and HP ChemStation for data collection and treatment. Normal-phase separations of the proanthocyanidin oligomers were performed under the conditions previously described by Hammerstone et al. (1999) [25]. Briefly, separations of the procyanidin oligomers were performed on a 5-m Luna silica column (250 × 4.6 mm) (Phenomenex, Torrance, CA, USA) at 37 °C using an injection volume of 10 L. The ternary mobile phase consisted of (A) dichloromethane, (B) methanol, and (C) acetic acid and water (0.01:1 *v*/*v*). Separations were achieved by a series of linear gradients of B in A with constant 4% C at a flow rate of 1 mL/min as follows: elution starting with 14% B in A; 14–28.4% B in A, 0–30 min; 28.4–50% B in A, 30–60 min; 50–86% B in A, 60–65 min; 65–70 min isocratic. Data were collected using both the UV detector (Agilent Technologies, Santa Clara, CA, USA), at 280 nm and the fluorescence detector (FLD) (Agilent Technologies, Santa Clara, CA, USA), at an excitation wavelength of 276 nm and an emission wavelength of 316 nm. Other FLD conditions included a photomultiplier tube gain of 12, frequency of 110 Hz, and response time of 2 s.

### 2.3. Numerical Simulation

**Figure 2:**
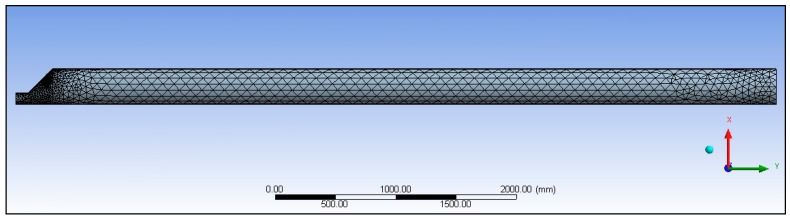
Finite volume model of the pipeline.

A numerical model was used to simulate the thermo-fluid-dynamic process that occurred inside the delivery pipeline between the liquid CO_2_ and the mashed grapes. The aim was to evaluate the *L*_0_ pipe length at which the temperature distribution became constant and the thermodynamic exchange between liquid CO_2_ and must was complete. Such an evaluation is important because after the pipe length *L*_0_, no liquid CO_2_ pockets should reach the cyclone. Liquid CO_2_ pockets that enter the cyclone will result in ice formation. The CFX 5.0 Ansys program code was used. Because of the pipe’s geometry, it was assumed to be symmetrical, and therefore only half of the delivery pipe was modelled, with approximately 4.0 × 10^5^ elements (Figure 2). 

The characteristics of the fluids used as inputs were as follows: liquid CO_2_ with a pressure of 20 × 10^5^ Pa and a temperature of −20.0 °C and grape juice as an aqueous solution at atmospheric pressure and a temperature of 37.0 °C. We performed the numerical simulation considering a final temperature equal to 10.0, 8.0, 6.0, or 4.0 °C, and we evaluated the pipe length after which the final temperature became uniform.

The injection of radial liquid CO_2_ in the inlet section was also considered, in addition to a pipeline length of 8 m and constant diameter of 30 cm. Moreover, for simplicity, the pipe was considered to be linear. In the inlet section, the two fluids (mashed grape and liquid CO_2_) are not yet mixed.

As soon as the two fluids leave the inlet section, the mixing immediately begins, together with the change in phase between the gaseous CO_2_ and dry CO_2_ and the thermal exchange among the three species. To ensure fast cooling of the mashed grapes and to minimize the loss of organoleptic substances and natural aromas in the final product, the thermal exchange must be completed in the shortest time possible [26,27]. The physical properties of the two fluids are shown in Table 1. The discretization of the numerical model with a mesh is shown in Figure 2, and the boundary conditions considered are listed in Table 2. Table 3 reports the values of the characteristic parameters for the 4 considered cases.

### 2.4. Winemaking Process

Samples of the cooled mashed grape treated at each temperature were collected to fill 3 stainless steel tanks of 100-L capacity and then stored under an inert and controlled environment of carbon dioxide gas. In total, 12 stainless steel tanks were used to store the batches of mashed Chardonnay grapes treated at 10.0, 8.0, 6.0 and 4.0 °C. The temperature was maintained constant for all tanks and monitored during the entire experimentation period. The grapes were subjected to skin maceration immediately after crushing at the pre-set temperature for 24 h. Potassium meta-bisulfite (120 mg/kg grape) was added and mixed with the grapes prior to pressing. All musts obtained after pressing were adjusted to pH 3.4 ± 0.1, centrifuged at 3000 rpm for 10 min, and inoculated with 2% (*w*/*v*) of a pure culture of the *Saccharomyces cerevisiae* (*cerevisiae* A) yeast strain. In all cases, fermentation started in the tanks and was maintained at 22.0 °C. Thus, the only difference among the treatments was the cryo-maceration temperature. Temperature and density were measured daily. Manual punching down was conducted twice a day by “pigéage” (a classic French wine-making method where the grapes are stomped down in open vats by foot). The wines were then strained off, and the mash was pressed. Malolactic fermentation was then induced by inoculation with *Oenococcus oeni* lactic acid bacteria. When the second fermentation was finished, 20 mg/L sulfur dioxide were added to the wines. The wines were allowed to stay for 4 weeks and then analyzed.

### 2.5. Physical-Chemical Analysis

Ethanol was quantified using the method of Crowell & Ough (1979) [28]. Reducing sugars, phenolic compounds, volatile acidity, pH and tartaric acids were determined using European Economic Community (EEC) official methods (1990) [29]. In this work, the polyphenolic content of the wines obtained was analysed by HPLC according to a method described by Hammerstone et al. (1999) [25].

### 2.6. Organic Acid Analysis

An isocratic high-performance liquid chromatographic (HPLC) method was used for the separation and quantitative analysis of the major organic acids of wines such as citric, tartaric, malic, lactic, and acetic acids. An Aminex HPX87-H strong cation-exchange resin column eluted with dilute sulfuric acid was used with UV monitoring at 210 nm. The sample was prepared by membrane filtration and removal of phenolic compounds with disposable reverse phase cartridges. The use of mobile phases with different pH values allowed the separation of compounds such as malic acid and fructose that normally coelute [30]. The results of the HPLC analysis were compared with data in the literature.

### 2.7. Total Phenolics Assay

This analytical procedure was performed as reported by Ivanova et al. (2011) [31]. For the determination of total phenols, the Folin-Ciocalteu method was used [32]. After appropriate dilution of samples, 1.00 mL of solution was transferred to a 10-mL volumetric flask, and 5 mL of distilled water was added. Then, 0.5 mL of Folin-Ciocalteu reagent was added, and the resultant solution was mixed. After 3 min, 1.5 mL of Na_2_CO_3_ solution (5 g/L) was added, and after mixing, the volumetric flask was filled to the mark with distilled water. The solution was incubated at 50.0 °C for 16 min and then cooled at room temperature; the absorbance of this solution was read at a wavelength of 765 nm using distilled water as a blank. The total phenol concentration was expressed as gallic acid equivalents per 1 g of fresh sample after the construction of a calibration curve in the range of 0–100 mg/L. All samples were analysed in triplicate.

### 2.8. Total Catechins Assay

The *p*-(dimethylamino)cinnamaldehyde (*p*-DMACA) method was used for the determination of total catechins expressed as catechin equivalents (mg/L). The reagent was prepared in methanol and HCl (4:1 *v*/*v*) and contained 1% (*w*/*v*) DMACA. After appropriate dilution of the sample, 1 mL of solution was transferred to a 10-mL volumetric flask, and three drops of glycerine and 5 mL of *p*-DMACA reagent were added. The volumetric flask was filled to the mark with methanol, and the solution was allowed to stand for 7 min. The absorbance was then read at a wavelength of 640 nm using methanol as a blank [33]. All samples were analysed in triplicate.

### 2.9. Colour Intensity, Hue, Colour Composition and Brilliance of the Wines

The colour intensity of the wine was determined by measuring its absorbance. The colour intensity mainly depends on the anthocyanin content and structure [34]. By using a 2-mm optical path, the absorbance values were determined as reported by Ivanova et al. (2010) [33].

### 2.10. Statistical Analysis

The data for each variable were analysed using multifactor analysis of variance (ANOVA). The statistical significance of each factor under consideration was calculated using the LSD test. The data were statistically analysed using Statgraphic Plus 5.1 software (Statpoint Technologies, Warrenton, VA, USA).

## 3. Results and Discussion

### 3.1. Numerical Simulation

**Figure 3:**
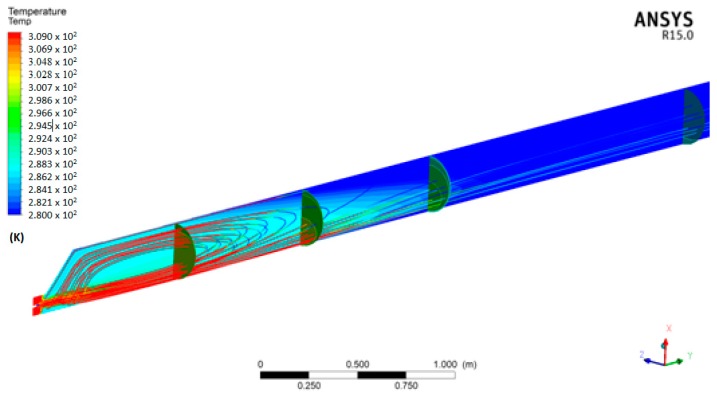
Temperature distribution in the median section of the pipeline and flow pattern-case 4, Table 3.

**Figure 4:**
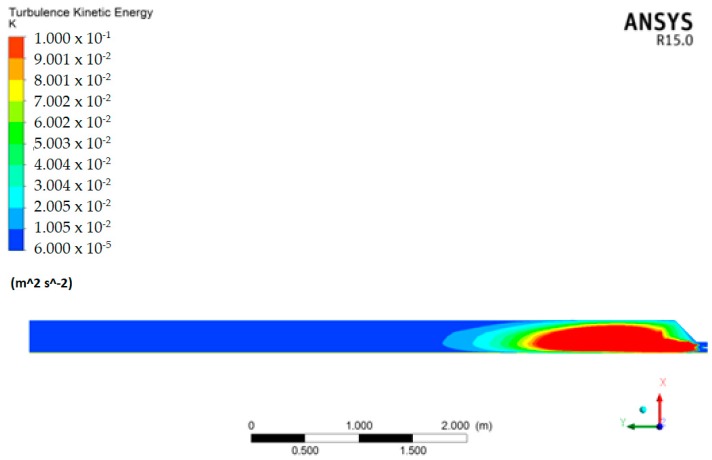
Turbulent kinetic energy distribution in the median section of the pipeline.

The results of the numerical simulation show that there is a zone with relevant temperature gradients and another zone in which these gradients are negligible (Figure 3 and Figure 4).

**Figure 5:**
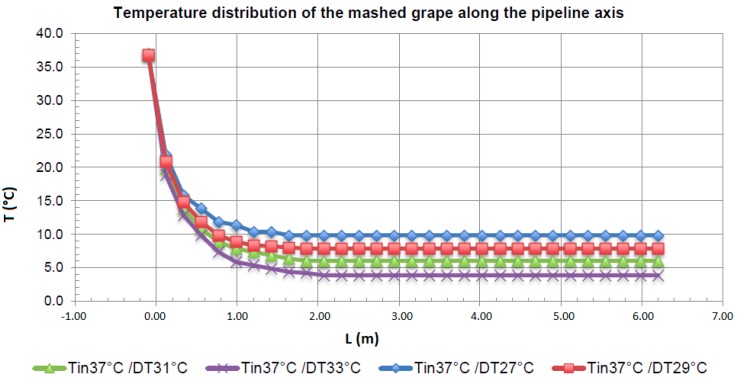
Temperature distribution of the mashed grape along the axis of the pipeline, for each case reported in the above Table 3 (*T_in_* (°C) = initial temperature of mashed grape; *T_out_* (°C) = Final temperature plant set up; DT = *T_in_ − T_out_*).

In particular, it was hypothesized that after the CO_2_ input, a turbulent regime was established in the mixture and after a length of pipe *L*_0_, measured starting from the input section, the temperature was uniform and equal to the pre-set temperature (*T*_out put_), taking into account that CO_2_ was inputted at 20 atm and therefore the mixed fluid mass underwent an acceleration (increasing the final flow velocity). In other words, by injecting liquid CO_2_, it cooled the liquid with which it came in contact, and generated a pipe length, *L*_0_, with non-uniform internal liquid temperature and with pockets of liquid CO_2_. The CO_2_ pockets vanished when the internal heat exchange inside the pipeline was exhausted, that is, after the pipe length *L*_0_. The value of *L*_0_ must be less than *L*_t_ (total length of the pipe), so as to avoid pockets of liquid CO_2_ arriving in the cyclone, which is located after pipe length *L*_t_ (Figure 4). The recirculation that appears in the first part of the pipeline due to the asymmetrical geometry of this part and the strong kinetics of the gaseous CO_2_, enhance the mixing phenomenon among the species involved, and consequently, thermal exchange. In all cases examined, the extinction length of this phenomenon, that is, the distance at which the temperature is approximately uniform, was approximately 2 m (Figure 5). The extinction length value is very important for avoiding the passage of liquid CO_2_ pockets into the cyclone and ice formation. Therefore, we must be certain that the liquid CO_2_ has completed thermal exchange before the cyclone zone, after the *L*_0_ length. In this case, *L*_0_ is approximately 2 m. Generally, it is necessary to verify that the total length of the pipeline *L*_t_ is greater than *L*_0_. In this case, the results guarantee that there are no CO_2_ liquid pockets in the cyclone, which can provoke ice formation.

### 3.2. Winemaking Process

The alcohol content in all of the produced wines ranged between 11.40 and 11.45 (*v*/*v*), and the titratable acidity values ranged between 6.00 and 6.09 g/L, expressed as tartaric acid. The volatile acidity values were approximately 0.30 ± 0.02 g/L, expressed as acetic acid. The pH values ranged between 3.19 and 3.26. These values are characteristic of young white table wines and are proof of a good winemaking process.

### 3.3. Chemical Pnalysis

The chemical analyses of wines made from grapes subjected to the cryo-maceration process at different temperatures did not reveal any significant differences in density and pH (Table 4).

By contrast, the polyphenol content increased as temperature decreased from 10.0 to 6.0 °C. However, a further decrease in temperature from 6.0 to 4.0 °C did not significantly increase the polyphenol content. All wines had high phenol values because of the low cryo-maceration temperatures used. In fact, a deeper yellow colour (OD 420) was a direct consequence of the higher phenolic content of the wines. Although catechins were not sensitive to the different cryo-maceration temperatures, one of their monomers, (−) epicatechin, exhibited higher levels at lower temperatures [35]. The means and standard deviations of the phenolic parameters showed significant differences when a lower cryo-maceration temperature was used. Furthermore, the use of lower cryo-maceration temperatures had a positive effect on the total concentration of pro-anthocyanins, which contribute to wine colour. Alcohol, reducing sugars and volatile acidity were not significantly different. The same considerations can be applied to the levels of total acidity and pH values. The maceration process was carried out in such a way that during this phase there were no fermentation processes. On the other hand, all wine producers know that fermentative processes do not take place at a temperature of 10.0 °C. Therefore, in all wineries, maceration is usually carried out with *T* at around 10.0 °C using different methods of cooling. Accordingly, the conventional fermentation used as a comparison test was wine obtained with a maceration temperature of 10.0 °C. In particular, the evaluation of the increase in polyphenols was only carried out according to the Folin-Ciocalteu assay. From these results, it could be deduced that by lowering the temperature from 10.0 to 8.0, and to 6.0 °C, a significant increase in polyphenols was obtained. However, a decrease from 6.0 to 4.0 °C did not provide further significant increases in polyphenols, and also implied a strong increase in energy costs. Moreover, these data show that the different cryo-maceration temperatures did not affect the fermentative activity of the yeasts. On the other hand, it is known that measurement of antioxidant activity using in vitro assays has become very popular over recent decades. Indeed, the measurement concentrations of total phenolics, flavonoids, and other compound (sub)classes using UV/Vis spectrophotometry enable rapid screening. For a complete characterization of the bioactive compounds, chromatographic techniques and/or in vitro and in vivo assays can be used, as recently reported by Granato et al. (2018) [36].

### 3.4. Wine Phenolic Composition and Colour Variables of the Wines

The concentrations of total phenolic compounds increase with decreasing cryo-maceration temperature down to 6.0 °C; further decreases in temperature to 4.0 °C have a negligible effect [37]. White wines have different phenolic compositions that are characteristic for each variety. However, the concentrations of the phenolic families in wines depend not only on the grape variety but also on additional factors, such as the climatic conditions, the enological practices and the storage conditions [38,39]. During bottle ageing of wine, modifications of the polyphenolic composition occur as a result of different transformations, such as oxidation processes, condensation and polymerization reactions. These reactions include direct reactions between anthocyanins and flavanols or reactions between anthocyanins and flavanols through ethyl bridges [40,41], whereby stable pigments are formed that stabilize the wine colour. All of these reactions are related to changes in colour and sensorial characteristics, such as the flavour, bitterness and astringency of the final wine. The analysed wines contained 233.5−440.4 mg/L total phenols, and the content of catechins was 18.3–18.9 mg/L; the (+)-catechin content was 3.39–3.43 mg/L; (−)-epicatechin was 20.5–22.9 mg/L; proanthocyanidins were 38–51 mg/L. The obtained results for the analysed wines were also in agreement with the data published by EEC methods (EEC regulation 2676/90 1990) [29].

### 3.5. Organic Acid Analysis

The statistical evaluation showed that the HPLC method for identifying and quantitating the major carboxylic acids in the wines was highly reproducible and reliable. The results of the HPLC analyses were compared with the data obtained by standard methods reported in the literature. The calculated content of carboxylic acids was in very good agreement with the values obtained by other standard methods, and the precision of the quantitation was comparable to those of traditional methods.

### 3.6. Polyphenol Analysis

The polyphenolic fingerprint might be a useful tool for the classification of wines [42,43,44]. In this work, the polyphenolic content of the wines was analysed by HPLC according to a method described by Hammerstone et al. (1999) [25]. Several authors have shown that principal component analysis of the polyphenolic fraction can separate wines according to variety. A variety of techniques have been used to analyse phenolic composition. HPLC is mainly used for this purpose, occasionally connected to an MS, but other techniques, such as NIR and UV/Vis, have also been successfully used [45,46]. Many authors have studied phenolic compounds in grapes and wines using HPLC as the most suitable analytical technique [47,48,49]. However, this technique is not available in wineries for routine analyses, whereas spectrophotometric methods, which are more affordable due to their lower costs, lower reagent consumption and rapid measurements, can be used for wine and grape analyses to follow the changes in polyphenol content during grape ripening and the winemaking process. The most commonly used methods are for the determination of the total phenolics and catechins [32].

### 3.7. Sensory Analysis

A sensory analysis using a consumer test was also conducted on the final product obtained to determine whether differences were highlighted in the perception and pleasantness of the products that had been processed (10.0, 8.0, 6.0, 4.0 °C). The consumer test showed that a group of regular consumers (not experts) of Chardonnay wine (16 individuals: 9 men and 7 women) whowere offered anonymous samples of the product successfully identified the quality of the samples, which they tasted using the scores described in the report table. For this number of tasters, the number of correct answers (i.e., the number of individuals correctly identifying which of the three samples was different) must be *p* < 0.10 for the results to be significant at the 90% confidence level (i.e., an error factor equal to 0.05). The tasters answered correctly for each parameter in an interval scale (0 best–100 worst) and responses were averaged. The average values were taken for statistical treatment and are reported in Table 5. Based on these results, we can conclude that the product obtained with the cryomaceration temperature near to 6.0 °C obtained the best result in the consumer test. However, the data obtained in the preference tests suggest that the samples obtained with a cryomaceration temperature of 6.0°C corresponded to the best taste (Table 5).

### 3.8. Statistical Analysis

Statistical analysis was performed for all data reported in Table 4 and Table 5 Mean values in different columns designated by different letters are significantly different by Fisher’s Test at *p* ≤ 0.005. Mean values in different columns designated by the same letter are not significantly different by Fisher’s Test at *p* ≥ 0.005.

## 4. Conclusions

In this work, different cooling temperatures were used during the cryo-maceration of Chardonnay grapes (10.0, 8.0, 6.0, 4.0 °C). The polyphenol quantity extracted at the different cryo-maceration temperatures was considered without phase changes that could affect the chemical and physical characteristics of the mashed grapes. In particular, lowering the cryo-maceration-temperature from 10.0 to 6.0 °C resulted in an increase in polyphenols. Below a temperature of 6.0 °C, further increases in polyphenols were negligible, whereas liquid CO_2_ consumption increased notably. For Chardonnay wine, lowering the cryo-maceration temperature below 6.0 °C did not significantly increase polyphenol content but increased liquid CO_2_ consumption. This increased consumption of liquid CO_2_ can not only increase the cost of wine production but also have a greater environmental impact. Although polyphenols have antioxidant properties, they can promote the browning of wine. Finally, a numerical simulation was used to evaluate the *L*_0_ pipe length after which the temperature is constant to ensure that there is no ice formation in the cyclone.

## Figures and Tables

**Table 1:** Characteristics of the fluids.

Properties	Grape Juice	Liquid CO_2_
Density (kg/m^3^)	1300	1032
Cp (J/kg K)	2710	2050
Thermal conductibility (w/m K)	0.61	0.132
Viscosity (kg/m s)	0.001003	0.00014

**Table 2:** Boundary conditions considered.

Conditions	Grape Juice	Liquid CO_2_	Mixture
Inlet	*V* = 1 m/s *T* = 310 K	*V* = 20 m/s *T* = 253 K	
Outlet	*T* = *T*_final_	*T* = *T*_final_	*p* = 1 atm
Wall	adiabatic	adiabatic	adiabatic

**Table 3:** Values of the characteristics parameters for the four considered cases *T*_out_ (°C) = Final temperature plant set up; *T*_in_ (°C) = initial temperature of mashed grape.

Case	*T*_in_ (°C)	*T*_out_ (°C)	DT (°C)	Mashed Grapes (kg/s)
1	37	10.0	27.0 ± 0.5	5.6
2		8.0	29.0 ± 0.5	
3		6.0	31.0 ± 0.5	
4		4.0	33.0 ± 0.5	

**Table 4:** Analytical determinations performed on wines made from grapes subjected to cryomaceration processes at different final temperatures (Chardonnay grape).

Parameters	*T*_out_ = 10.0 °C	*T*_out_ = 8.0 °C	*T*_out_ = 6.0 °C	*T*_out_ = 4.0 °C
Alcohol (%) (*v*/*v*)	11.43 ± 0.30 ^a^	11.42 ± 0.10 ^a^	11.41 ± 0.20 ^a^	11.40 ± 0.10 ^a^
Density 20.0 °C	0.9901 ± 0.002 ^a^	0.9901 ± 0.001 ^a^	0.9901 ± 0.010 ^a^	0.9901 ± 0.003 ^a^
Titratable acidity (g/L)	6.05 ± 0.05 ^a^	6.03 ± 0.02 ^a^	6.00 ± 0.03 ^a^	6.00 ± 0.01 ^a^
Volatile acidity (g/L)	0.30 ± 0.02 ^a^	0.31 ± 0.01 ^a^	0.30 ± 0.01 ^a^	0.3 ± 0.02 ^a^
Total extract (g/L)	21.00 ± 0.48 ^a^	21.90 ± 0.51 ^a^	22.70 ± 0.46 ^a^	22.73 ± 0.59 ^a^
pH	3.20 ± 0.06 ^a^	3.23 ± 0.05 ^a^	3.25 ± 0.02 ^a^	3.26 ± 0.03 ^a^
OD 420 nm	0.11 ± 0.01 ^a^	0.15 ± 0.03 ^a^	0.162 ± 0.04 ^a^	0.163 ± 0.02 ^b^
OD 320 nm	6.9 ± 0.72 ^a^	7.7 ± 0.53 ^a^	8.4 ± 0.77 ^a^	8.5 ± 0.54 ^b^
OD 280 nm	8.5 ± 0.74 ^a^	9.3 ± 0.67 ^a^	10.8 ± 0.75 ^a^	10.9 ± 0.69 ^b^
Total phenols (mg/L)	233.5 ± 13.5 ^a^	336.4 ± 12.7 ^a^	438.4 ± 14.7 ^a^	440.4 ± 12.5 ^b^
Catechins (mg/L)	18.3 ± 0.24 ^a^	18.5 ± 0.32 ^a^	18.7 ± 0.24 ^a^	18.9 ± 0.33 ^b^
(+)-Catechin (mg/L)	3.39 ± 0.02 ^a^	3.40 ± 0.01 ^a^	3.42 ± 0.03 ^a^	3.43 ± 0.01 ^b^
(−)-Epicatechin (mg/L)	20.5 ± 3.7 ^a^	21.3 ± 2.9 ^a^	22.5 ± 3.9 ^a^	22.9 ± 2.8 ^b^
Proanthocyanidins (mg/L)	38 ± 2 ^a^	46 ± 5 ^a^	50 ± 3 ^a^	51 ± 4 ^b^

OD optical density; (Mean value ± dxm = standard deviation of the mean). Analysis of variance (ANOVA): mean values within different columns designated by different letters are significantly different by the Fisher’s tests at *p* ≤ 0.005; mean values within different columns designated by the same letters are not significantly different by the Fisher’s tests at *p* ≤ 0.005.

**Table 5:** Sensory analysis: scores range from 1 to 100, in which the value 100 corresponds to the lowest quality.

Treated Grape	Visual	Smelling	Gustatory	Harmony	Scores
Chardonnay 10.0 °C	6.7 ± 1.2 ^b^	13.2 ± 2.5 ^b^	19.0 ± 1.5 ^b^	8.0 ± 2.0 ^b^	46.3 ± 4.7
Chardonnay 8.0 °C	5.9 ± 1.1 ^c^	11.3 ± 2.6 ^c^	18.4 ± 1.7 ^c^	9.9 ± 2.4 ^c^	43.9 ± 6.9
Chardonnay 6.0 °C	5.1 ± 1.3 ^d^	10.1 ± 2.7 ^b^	17.1 ± 1.6 ^c^	9.1 ± 2.6 ^d^	41.4 ± 6.7
Chardonnay 4.0 °C	5.2 ± 1.1 ^d^	10.2 ± 2.6 ^b^	17.2 ± 1.8 ^c^	9.1 ± 2.5 ^d^	41.7 ± 6.9

Analysis of variance (ANOVA): mean values within different columns designated by different letters are significantly different by Fisher’s tests at *p* ≤ 0.005; mean values within different columns designated by the same letters are not significantly different by Fisher’s tests at *p* ≤ 0.005.
